# MAEL contributes to gastric cancer progression by promoting ILKAP degradation

**DOI:** 10.18632/oncotarget.22970

**Published:** 2017-12-06

**Authors:** Xing Zhang, Yichong Ning, Yuzhong Xiao, Huaxin Duan, Guifang Qu, Xin Liu, Yan Du, Dejian Jiang, Jianlin Zhou

**Affiliations:** ^1^ Key Laboratory of Protein Chemistry and Developmental Biology of The Ministry of Education, College of Life Science, Hunan Normal University, Changsha 410081, Hunan, China; ^2^ College of Biology, Hunan University, Changsha 410082, Hunan, China; ^3^ The First Affiliated Hospital, Hunan Normal University, Changsha 410005, Hunan, China; ^4^ Hunan Key Laboratory of Pharmacodynamics and Safety Evaluation of New Drugs, Changsha 410331, Hunan, China

**Keywords:** MAEL, gastric cancer, ILKAP, CHK1, p38 MAPK

## Abstract

The cancer-testis gene *MAEL* is involved in the development and progression of bladder, liver and colorectal cancers. However, its role in other cancers is unclear. By systematically analyzing transcriptomics and genomics data from various cancer databases, we identified that the *MAEL* gene is aberrantly elevated in gastric cancer (GC) tissues and that its expression is strongly negatively correlated with DNA methylation (Pearson's correlation coefficient = −0.675). Survival analysis revealed that *MAEL* expression may serve as a prognostic marker for GC patients (overall survival: hazard ratio [HR] = 1.54, *p* = 1.2E-4; first progression: HR = 1.51, *p* = 8.7E-4). *In vitro* and *in vivo* experiments demonstrated that silencing *MAEL* expression in the GC cell lines HGC-27 and AGS inhibits proliferation, colony formation, migration, invasion and growth of xenograft tumors, whereas *MAEL* overexpression exerts the opposite effects in the normal gastric cell line GES-1. Mechanistically, MAEL promotes the lysosome-dependent degradation of the protein phosphatase ILKAP, leading to increased phosphorylation of its substrates (p38, CHK1 and RSK2). Moreover, adenovirus-mediated *ILKAP* overexpression reversed the oncogenic effects of *MAEL in vitro* and *in vivo.* Taken together, these results indicate that *MAEL* exerts its oncogenic function by promoting ILKAP degradation in the GC.

## INTRODUCTION

Gastric cancer (GC) is the fifth most frequently diagnosed cancer and the third leading cause of cancer-related death worldwide [1]. The etiology of GC is very complicated; gastric carcinogenesis is related to *Helicobacter pylori* infection, genetic alterations, and dietary and other risk factors [2]. Even though many advances in the treatment of GC have been achieved, the 5-year survival rate of GC is still less than 30% [2]. Tumor heterogeneity and the lack of effective therapeutic targets are the two main obstacles for precision GC medical treatments [3]. Therefore, it is urgently necessary to identify novel diagnostic, predictive, and prognostic biomarkers for targeted therapies.

Human cancers frequently exert soma-to-germline transformations by reactivating genes that are normally required for germline stemness, fitness, or longevity [4–6]. These genes, which are only expressed in germ cells and are aberrantly expressed in some cancer cells, are referred to as cancer-germline or cancer-testis genes [5]. It is commonly believed that DNA hypomethylation contributes to the abnormal activation of most cancer-germline genes in somatic tissues [5]. The Maelstrom (*MAEL*) gene was first cloned in *Drosophila* [7], and it plays essential roles in meiosis and spermatogenesis [8, 9]. Our previous study demonstrated that *MAEL* is a cancer-testis gene that is only expressed in spermatocytes, round and early elongating spermatids in the testis, but is activated by demethylation in breast cancer cells [10]. Abnormal activation of *MAEL* is also found in liver, colon and bladder cancers and contributes to tumorigenesis, cancer cell survival, invasion and epithelial-mesenchymal transition (EMT) [11–14]. However, in contrary to what has been observed in the above cancer types, *MAEL* exerts a repressive effect on cell invasiveness in ovarian cancer [15]. The roles of *MAEL* in GC progression and development have not been investigated.

With the advances in high-throughput technologies (including microarrays and next-generation sequencing) and computational techniques, cancer research has been brought into a “big data” era. Currently, there are numerous high-throughput datasets related to cancer that are freely available in public data repositories, such as TCGA (The Cancer Genome Atlas), GEO (Gene Expression Omnibus) and EGA (European Genome-phenome Archive). These “big data” have greatly facilitated the systematic analysis of the key genes and pathways involved in tumorigenesis [16]. Here, we used cancer genomics and transcriptomics data to systematically investigate the expression and clinical significance of *MAEL*. We found that *MAEL* was significantly overexpressed in GC tissues and that high *MAEL* mRNA levels predict poor survival in GC patients. Moreover, we demonstrated the oncogenic roles and underlying mechanisms of *MAEL* in gastric cancer.

## RESULTS

### *MAEL* mRNA is overexpressed and predicts poor survival in gastric cancers

**Figure 1:**
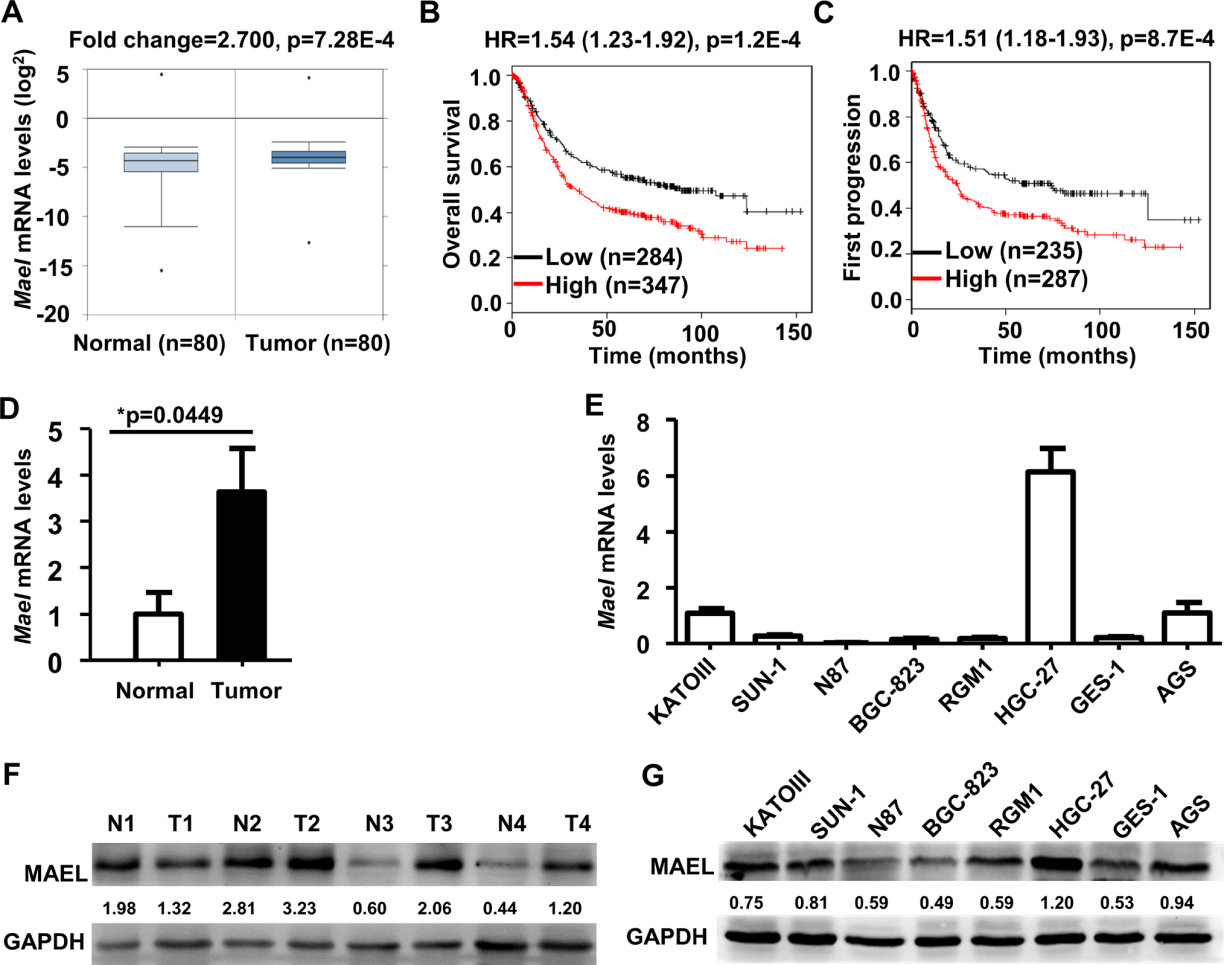
Expression and prognostic significance of *MAEL* gene in gastric cancer (**A**) Differential expression of *MAEL* mRNA between gastric normal and tumor tissues were analyzed by Onocomine based on the NCBI GEO dataset GSE27342. (**B**, **C**) Correlation of *MAEL* mRNA and overall/first-progression survival in gastric cancer was calculated using Kaplan Meier plotter based on the NCBI GEO datasets (GSE14210, GSE15459, GSE22377, GSE29272, GSE51105, and GSE62254). HR: hazard ratio, n: sample size. (**D**, **E**) The *MAEL* mRNA expression in the paired gastric tumor and adjacent normal tissues (**D**) and cell lines (**E**) were detected by realtime PCR. The *MAEL* mRNA expression levels were normalized using GAPDH control, and expressed as the mean ± standard deviation (S.D) of three replicates. (**F**, **G**) The MAEL protein levels in the paired gastric tumor and adjacent normal tissues (**F**) and cell lines (**G**) were detected by Western blotting. Staining intensity was quantitated by Image J software. The number below the blots indicates relative band intensity of MAEL protein normalized against that of GAPDH. N: normal adjacent noncancerous tissue, T: tumor tissue.

The cancer microarray database on the Oncomine platform was searched to analyze the differential expression of *MAEL* mRNA between cancerous and noncancerous tissues. The results showed that the levels of *MAEL* mRNA in gastric cancer (GC) (GEO: GSE27342 [17]), glioblastoma (GEO: GSE4536 [18]), invasive breast cancer (GEO: GSE9014 [19]) and lung adenocarcinoma (GEO: GSE31210 [20]) were significantly higher than their corresponding normal tissues (fold change ≥2, *p*-value < 0.01) (Figure 1A and Supplementary Figure 1A–1C) and that *MAEL* ranked in the top 6%, 10%, 16% and 20% of overexpressed genes in gastric, brain, breast and lung cancers, respectively.

The impact of *MAEL* expression on survival was investigated using Kaplan-Meier Plotter, which includes the gene expression data and survival information from the breast, ovarian, lung and gastric cancer patients [21]. The results revealed that higher *MAEL* mRNA expression was significantly associated with a shorter overall survival (OS) (hazard ratio [HR] = 1.54, 95% confidence interval [CI]: 1.23–1.92, logrank *p*-value [*p*] = 1.2E–4) and first progression survival (FP) (HR = 1.51, CI: 1.18–1.93, *p* = 8.7E-4) in gastric cancer patients (Figure 1B and 1C). However, there was no significant correlation between *MAEL* expression and the OS of patients with breast (HR = 0.89, C CI: 0.69–1.27, *p* = 0.53), lung (HR = 1.07, CI: 0.9–1.26, *p* = 0.45) or ovarian (HR = 1.02, CI: 0.82–1.28, *p* = 0.85) cancer (Supplementary Figure 1D–1F). These results indicated that *MAEL* expression in GC tissues is an unfavorable prognostic marker for GC patients.

According to the above results of meta-analysis, we were interested in the roles of *MAEL* gene in the GC. Therefore, we verified *MAEL* mRNA expression using real time PCR and MAEL protein level using Western blotting in 4 pairs of gastric tumor and adjacent normal tissues and 8 gastric cell lines. In consistent with the result of Oncomine analysis (Figure 1A), the average level of *MAEL* mRNA in gastric tumor tissues was significantly higher than that in the adjacent noncancerous tissues (Figure 1D); MAEL protein levels were also higher in the cancer tissues compared to the noncancerous tissues in 3 out of 4 of the tissue pairs investigated (Figure 1F). In gastric cell lines, the cancer cells HGC-27, AGS and KATOIII displayed significantly higher levels of both mRNA and protein than the normal cells GES-1 and RGM1 (Figure 1E and 1G).

### *MAEL* expression is induced by hypomethylation in gastric cancers

**Figure 2:**
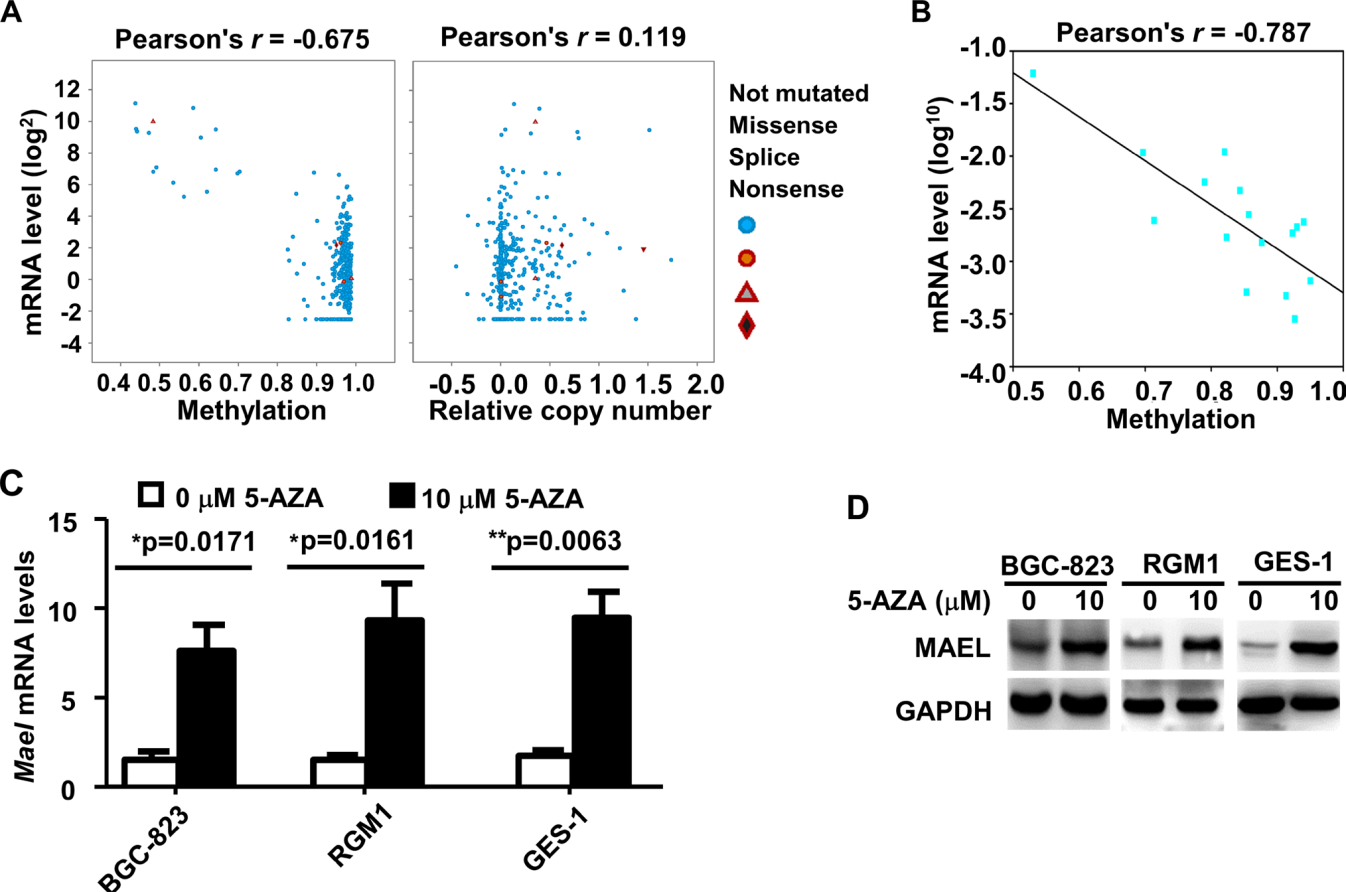
Expression of *MAEL* gene is regulated by promoter methylation in gastric cancer (**A**) Correlation between *MAEL* expression and DNA methylation/gene copy number were analyzed by cBioPortal based on the TCGA data derived from 478 gastric adenocarcinoma patients. (**B**) Correlation between *MAEL* expression and DNA methylation in the 4 pairs of gastric tumor and adjacent normal tissues and 8 gastric cell lines. The DNA methylation of *MAEL* gene promoter was detected by pyrosequencing assay, and the correlation between methylation and mRNA expression (in Figure 1D and 1E) was calculated and plotted by SigmaPlot (Systat Software, San Jose, CA). (**C**, **D**) Effects of treatment with 5-AZA on the levels of *MAEL* mRNA and protein. Cells were treated with or without 5-AZA for 72 h, and then harvested for isolation of mRNA and protein. The levels of *MAEL* mRNA and protein were detected by realtime PCR and Western blotting, respectively.

To investigate whether *MAEL* expression was regulated by gene amplification or DNA methylation, we analyzed the correlation between DNA variation and mRNA expression using cBioPortal based on the Stomach Adenocarcinoma data (TCGA, Provisional). The rates of DNA mutation and copy number variation are 2.4% and 1.8%, respectively, among the 478 gastric adenocarcinoma tissue cases. The *MAEL* mRNA levels are weakly correlated with the *MAEL* gene copy number (Pearson’s correlation coefficient (*r*) = 0.119) and strongly negatively correlated with methylation of CpG island in the *MAEL* gene promoter (Pearson’s *r* = −0.675) (Figure 2A). We verified the CpG island methylation of *MAEL* gene promoter among 4 pairs of gastric tumor and adjacent normal tissues and 8 gastric cell lines using pyrosequencing (Figure 2B and Supplementary Figure 2). Pearson’s correlation analysis was used to analyze the correlation between CpG methylation level and mRNA expression, and revealed a significantly negative relationship (Pearson’s *r* = −0.787) (Figure 2B). This result is consistent with that of cBioPortal analysis. Additionally, we treated the cell lines (BGC-823, RGM1 and GES-1) with the demethylating agent 5-Aza-2-Deoxycytidine (5-AZA), which significantly upregulated levels of both *MAEL* mRNA and protein (Figure 2C and 2D), suggesting that DNA hypomethylation in the *MAEL* gene promoter possibly contributes to its overexpression in GC cells.

### *MAEL* knockdown inhibits gastric cancer progression

**Figure 3:**
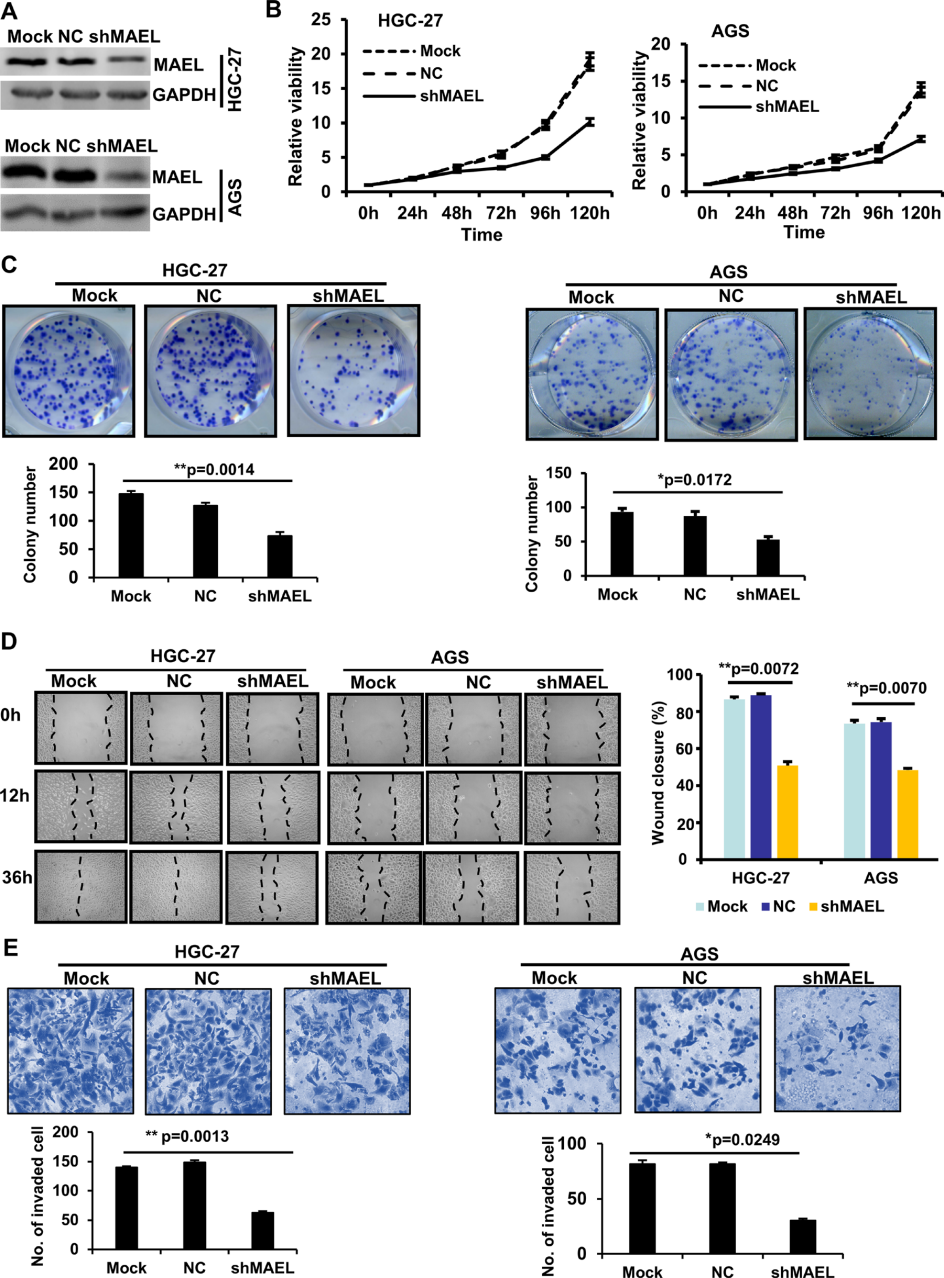
*MAEL* knockdown inhibits proliferation, migration and invasion in HGC-27 and AGS GC cells (**A**) MAEL protein levels in the untreated GC cells (mock) and the GC cells with stably expressing *MAEL* shRNA (shMAEL) or scrambled shRNA (NC). (**B**) The cell growth was determined by MTT assay. (**C**) Representative images of colony (upper panel) and the colony numbers (lower panel). (**D**) Representative images (left panel) of wound area at the indicated time and percentage of wound closure at 36 h after scratching (right panel). (**E**) Representative images (upper panel) and statistical analysis (lower panel) of invaded cells in the Transwell invasion assay. All the values were presented as means ± S.D for at least three independent *in vitro* experiments. Differences between two groups were analyzed by student *t*-test; ^*^p-value ≤ 0.05 and ^**^p-value ≤ 0.01.

**Figure 4:**
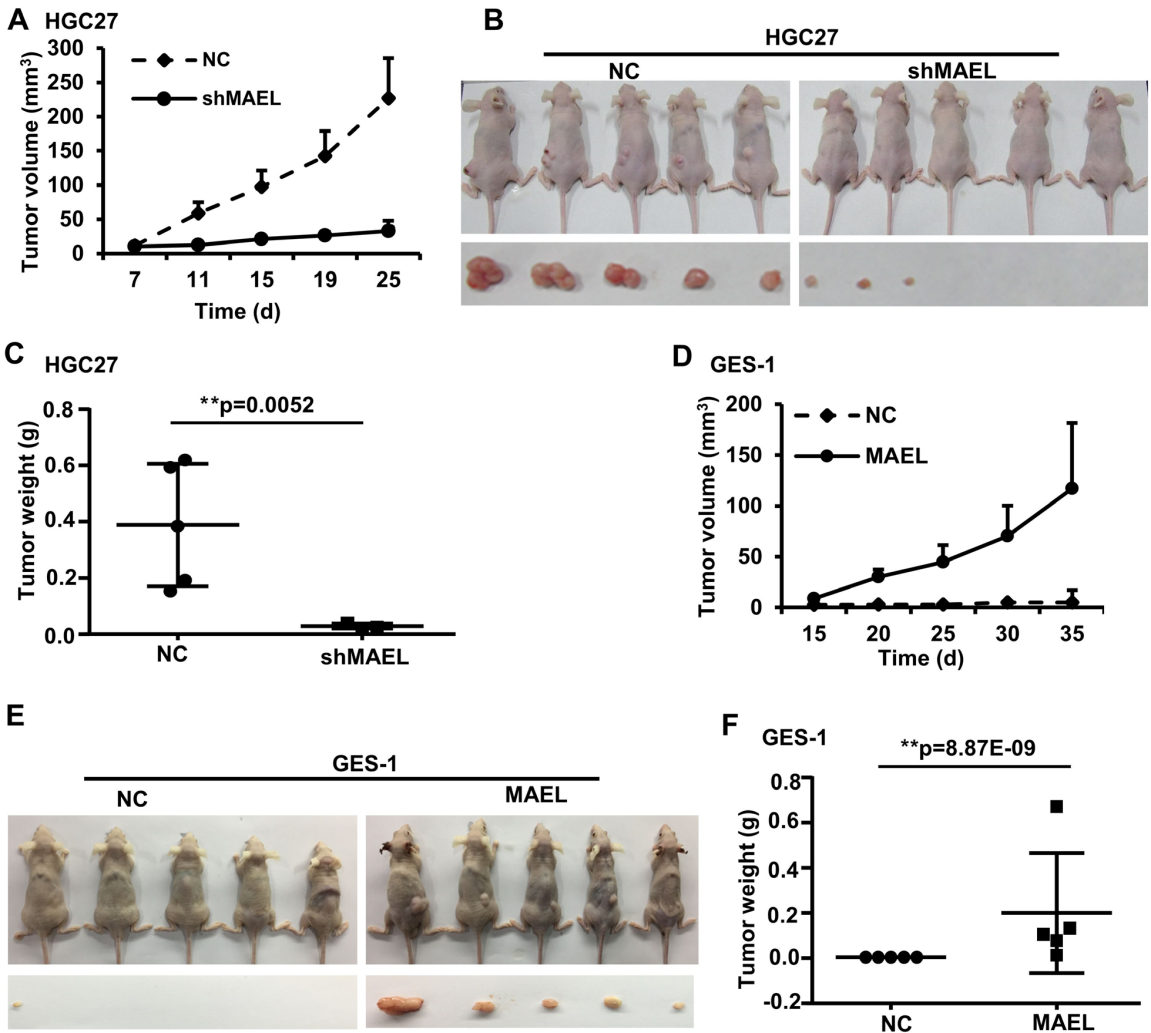
The influence of *MAEL* on the growth of xenograft tumors (**A**–**C**) *MAEL* knockdown in HGC-27 cell inhibits the growth of xenograft tumors. BALB/c nude mice were injected with 5 × 10^6^ HGC-27 cells expressing scrambled shRNA (NC) or MAEL shRNA (shMAEL). (**A**) Dynamic volume (mean ± S.D, *n* = 5) of xenograft tumors at different time after injection. (**B**) Images of the nude mice and their xenograft tumors at 30 d after injection. (**C**) Weight (mean ± SD, *n* = 5) of xenograft tumors at 30 d after injection. (**D**–**F**) *MAEL* overexpression in GES-1 cell promotes the growth of xenograft tumors; BALB/c nude mice were injected with 1 × 10^7^ GES-1 cells expressing empty vector (NC) or *MAEL* cDNA. (**D**) Dynamic volume (mean ± SD, *n* = 5) of xenograft tumors at different time after injection. (**E**) Images of the nude mice and their xenograft tumors at 40 d after injection. (**F**) Weight (mean ± SD, *n* = 5) of xenograft tumors at 40 d after injection. Differences between two groups were analyzed by student *t*-test, ^*^p-value ≤ 0.05 and ^**^p-value ≤ 0.01.

To investigate the role of *MAEL* in GC cells, we used a lentivirus-delivered shRNA system to generate stable GC cell lines (HGC-27 and AGS) that express scrambled or *MAEL* shRNA (Figure 3A). MTT assay revealed that *MAEL* knockdown inhibited cell proliferation in both AGS and HGC-27 cells (Figure 3B). In a colony formation assay, fewer colonies were observed in the stable AGS and HGC-27 cells, whose *MAEL* expression was inhibited compared with mock cells or cells infected with the empty lentivirus vector (Figure 3C). The wound-healing assay showed that the *MAEL*-silenced cells demonstrated slower closure of the scratched wound compared with the control cells, indicating that *MAEL* depletion inhibits cell migration (Figure 3D). In the transwell invasion assay, we observed that the number of invaded cells decreased when *MAEL* expression was suppressed by shRNAs (Figure 3E). Silencing *MAEL* expression in SUN-1 cells also inhibited cell growth, colony formation, wound healing and invasion (Supplementary Figure 3). These results demonstrated that *MAEL* participates in GC cell proliferation, migration and invasion. Additionally, we further investigated the *in vivo* effects of *MAEL* on tumor growth using a subcutaneous xenograft tumor mouse model. As shown in Figure 4A–4C, the tumors derived from *MAEL*-depleted HGC-27 cells grew significantly slower than tumors derived from control cells (Figure 4A); at the termination of the experiment, the average weights of the tumors derived from *MAEL*-depleted HGC-27 cells was much less than the controls (Figure 4B and 4C). Conversely, *MAEL* overexpression in GES-1 cells promotes the formation and growth of xenograft tumors (Figure 4D–4F).

### MAEL interacts with ILKAP and promotes its lysosome-dependent degradation

**Figure 5:**
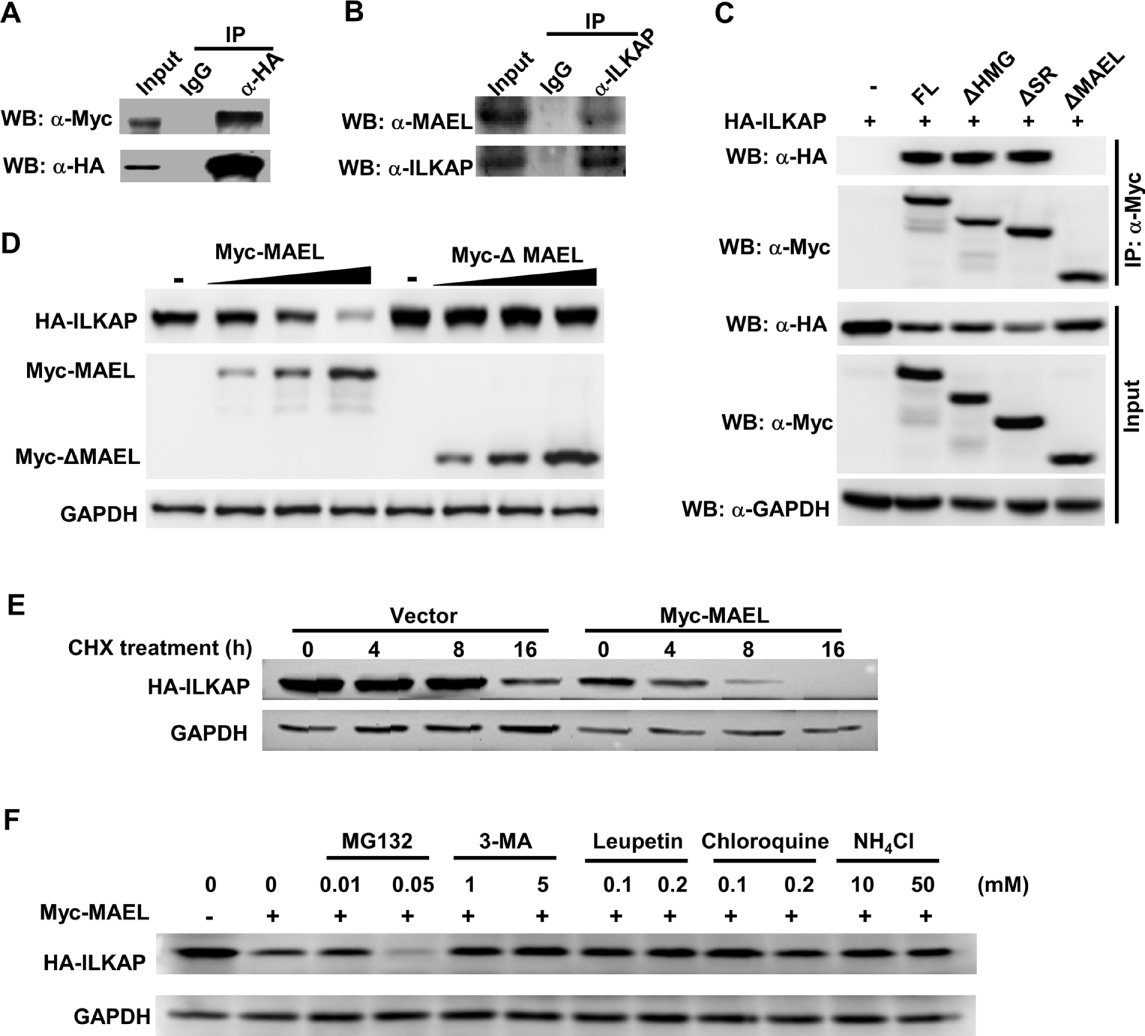
MAEL protein interacts with ILKAP protein and promotes its lysosomal-dependent degradation (**A**) Co-IP assay in HEK293 cells transiently transfected with HA-ILKAP and Myc-MAEL. Cell lysates (800 μg) were precipitated with anti-HA antibody or control IgG, and the immune complexes were subjected to Western blotting with anti-HA or anti-Myc antibody. Input is equivalent to 10% of the lysate used for the co-IP. (**B**) Co-IP of endogenous MAEL and ILKAP proteins in AGS cells. Cell lysates (800 μg) from AGS were precipitated with anti-ILKAP antibody or control IgG, and the immune complexes were subjected to Western blotting with anti-MAEL and anti-ILKAP antibody. Input is equivalent to 10% of the lysate used for the co-IP. (**C**). Co-IP assay in HEK293 cells transiently transfected with HA-ILKAP and Myc-tagged deletion mutant of *MAEL* gene. Cell lysates (800 μg) were precipitated with anti-Myc antibody, and the immune complexes were subjected to Western blotting with anti-HA or anti-Myc antibody. Input is equivalent to 10% of the lysate used for the co-IP. FL: full length MAEL, ΔHMG: HMG domain (aa 5–72) deletion, ΔSR: Ser-rich domain (aa 353–434) deletion, ΔMAEL: MAEL domain (aa 130–326) deletion. (**D**) *MAEL* overexpression decreases the ILKAP protein levels. HEK293 cells were co-transfected with a constant amount of HA-ILKAP plasmid and increasing amount of Myc-MAEL or Myc-ΔMAEL plasmid, cell lysates were prepared and subjected to Western blotting using anti-HA and anti-Myc antibodies at 24 h post-transfection. (**E**) *MAEL* overexpression decreases ILKAP stability. The HEK293 cells were co-transfected with HA-ILKAP and Myc-MAEL or empty vector and incubated with cycloheximide (CHX, 50 μg/ml) for different lengths of time, the cell lysates were subjected to Western blotting using anti-HA and anti-Myc antibodies. (**F**) Treatment with lysosome/autophagy inhibitor rescues ILKAP levels. The HEK293 cells were transfected with HA-ILKAP and Myc-MAEL and treated with CHX plus proteasome/lysosome inhibitors for 4 h, HA-ILKAP protein levels were detected by Western blotting using anti-HA antibody.

In our previous study, ILKAP was identified to be a component of the MAEL protein complex [22]. To confirm the protein interaction between MAEL and ILKAP, we performed co-immunoprecipitation (co-IP). We co-transfected a Myc-tagged *MAEL* expression plasmid and an HA-tagged *ILKAP* expression plasmid into HEK293 cells. At 24 h post-transfection, cell lysates were prepared and immunoprecipitated with anti-HA antibody or pre-immune IgG. Western blot assay showed that MAEL could be detected in the immune complex precipitated with anti-HA antibody, but not in complexes precipitated with IgG (Figure 5A). Moreover, co-IP experiment of endogenous MAEL and ILKAP proteins in AGS cells showed that endogenous MAEL protein could be precipitated efficiently by anti-ILKAP antibody, but not by IgG (Figure 5B). These results suggested that MAEL can interact with ILKAP.

To map the interaction site, we performed co-IP experiments using the HEK293 cells co-transfected with HA-ILKAP and Myc-tagged deletion mutants of MAEL. The results (Figure 5C) showed that deletion of the MAEL domain of MAEL protein disrupted the interaction between MAEL and ILKAP, suggesting that the MAEL domain is responsible for interaction with ILKAP. Interestingly, we found that ILKAP protein level in the cells co-transfected with HA-ILKAP and Myc-*MAEL* expression plasmid was much lower than that in the cells transfected with HA-ILKAP and empty vector, suggesting that MAEL decreases ILKAP protein levels. Then, we transfected a specific amount of the HA-ILKAP plasmid and increasing amounts of wild-type or MAEL domain deleted *MAEL* expression plasmid into HEK293 cells, and we found that increasing wild-type *MAEL* expression significantly decreased ILKAP protein levels, but MAEL domain deleted mutant did not affect ILAKP protein levels, indicating that MAEL mediated ILKAP degradation is protein-protein interaction dependent (Figure 5D). Thus, the effect of MAEL on ILKAP protein turnover was further investigated. The results revealed that ILKAP degradation occurred much more rapidly in the cells transfected with the Myc-MAEL plasmid than the empty vector (Figure 5E). To assess which pathway may affect ILKAP stability, we treated the cells with proteasome, lysosome or autophagy inhibitor. We observed that MAEL-induced ILKAP degradation could be rescued by the lysosome inhibitors Leupeptin, Chloroquine and NH4Cl and the autophagy inhibitor 3-MA, but not by the proteasome inhibitor MG132, indicating that ILKAP is degraded through an autophagy/lysosome-mediated pathway (Figure 5F).

### MAEL stimulates p38 MAPK, CHK1 and RSK2 phosphorylation by inhibiting ILKAP

**Figure 6:**
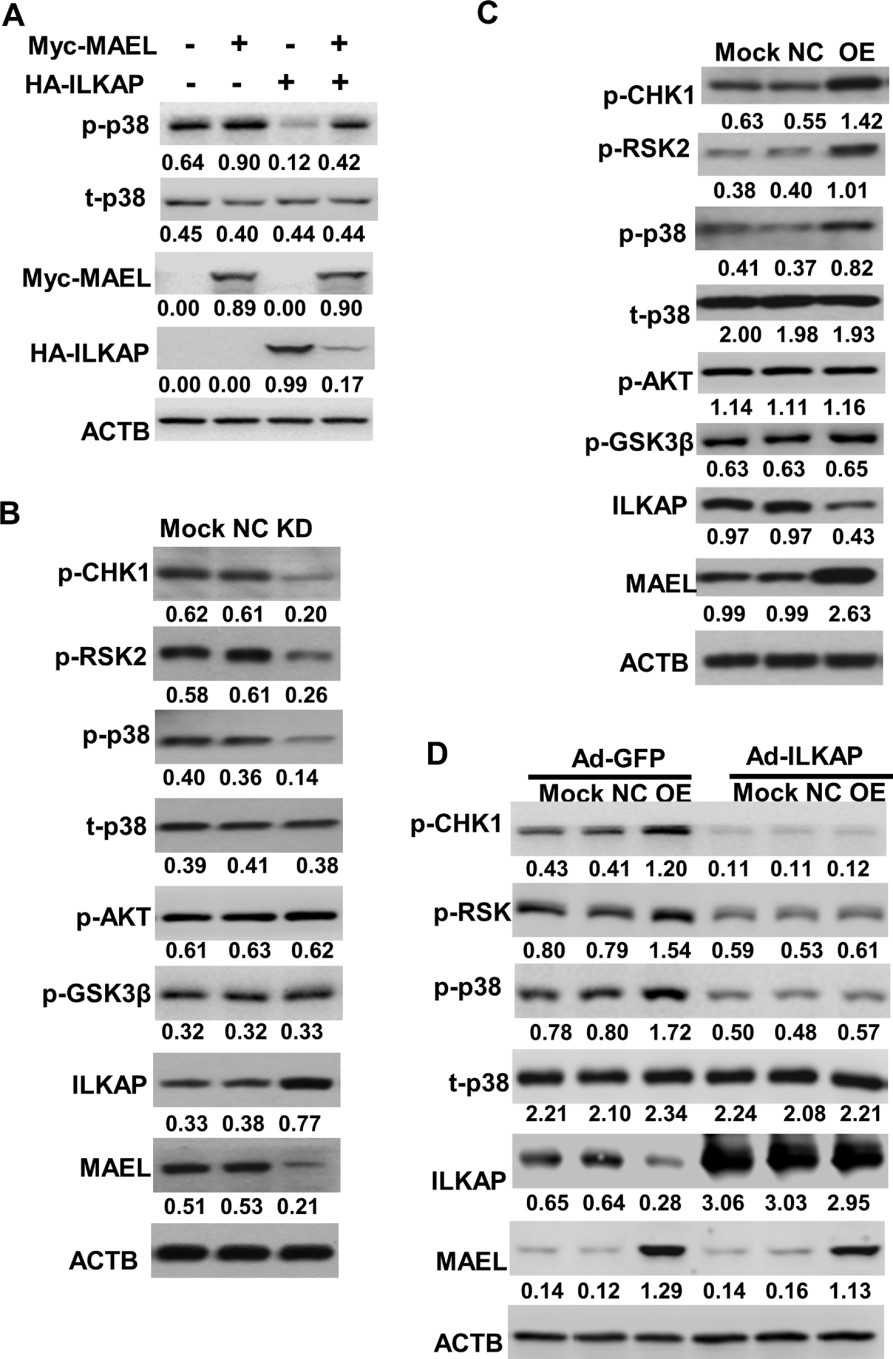
MAEL suppresses ILKAP-mediated dephosphorylation (**A**) *MAEL* overexpression increases while ILKAP overexpression decreases p38 phosphorylation in HEK293 cells. HEK293 cells were transiently transfected with Myc-MAEL or/and HA-ILKAP expression plasmid. At 24 h post-transfection, the phosphorylation of p38 was detected using Western blot with anti-phospho-p38 (T180/T182) antibody. (**B**) Western blot shows that *MAEL* knockdown increases ILKAP protein levels but suppresses the phosphorylation of its substrates in HGC-27 cells. Mock: uninfected HGC-27 cells, NC: HGC-27 cells stably expressing scrambled shRNA, KD: HGC-27 cells stably expressing *MAEL* shRNA. (**C**) *MAEL* overexpression decreases ILKAP protein levels but increases the phosphorylation of its substrates in GES-1 cells. Mock: GES-1 cells, NC: GES-1 cells stably expressing empty vector, OE: GES-1 cells stably expressing *MAEL* cDNA. (**D**) Adenovirus-mediated *ILKAP* overexpression suppresses MAEL-induced phosphorylation of ILKAP substrates in GES-1 cells. The adenovirus expressing *GFP* (Ad-GFP) or *ILKAP* (Ad-ILKAP) was infected into the uninfected GES-1 cells (Mock), GES-1 cells stably expressing empty vector (NC) or *MAEL* cDNA (OE). At 48 h post-infection, Western blotting was performed to detect phosphorylation of ILKAP substrates. Staining intensity was quantitated by Image J software. The number below the blots indicates relative band intensity of MAEL protein normalized against that of ACTB.

The above results showed that MAEL promoted ILKAP degradation. ILKAP is a member of the serine/threonine phosphatase 2C (PP2C) family [23]. Therefore, we wanted to know whether MAEL affects the phosphorylation of ILKAP substrates. p38 MAPK (p38), CHK1 and RSK2 have been identified as potential ILKAP substrates [24]. First, we transfected HA-ILKAP plasmid with or without Myc-MAEL into HEK293 cells and found that *MAEL* overexpression augmented phosphorylated p38 (at Thr180/Thr182) levels, whereas *ILKAP* overexpression inhibited p38 phosphorylation (Figure 6A). Further study showed that MAEL depletion increased ILKAP protein levels and decreased p38, CHK1 (at Ser345) and RSK2 (at Ser227) phosphorylation in HGC-27 cells (Figure 6B), while MAEL overexpression exerted the opposite effects on ILKAP and its substrates in GES-1 cells (Figure 6C). In the gastric tissues and cell lines, ILKAP appeared to have an opposite expression pattern to MAEL and the phosphorylated CHK1, RSK2 and p38 (Supplementary Figure 4A and 4B). Moreover, in *MAEL*-overexpressing GES-1 cells, the increased phosphorylation of p38, CHK1 and RSK2 by *MAEL* overexpression was almost completely abrogated by adenovirus-mediated *ILKAP* overexpression (Figure 6D). Taken together, these results indicated that ectopic expression of *MAEL* contributes to increased p38, CHK1 and RSK2 phosphorylation by inhibiting ILKAP in gastric cancer cells.

ILKAP has been proven to selectively inhibit ILK-mediated GSK3β phosphorylation on Ser9, though it does not affect AKT phosphorylation [25, 26]. However, we did not observe significant effects of *MAEL* expression on either AKT (at Ser473) or GSK3β (at Ser9) phosphorylation (Figure 6B and 6C).

### *ILKAP* suppresses the oncogenic role of *MAEL*

**Figure 7:**
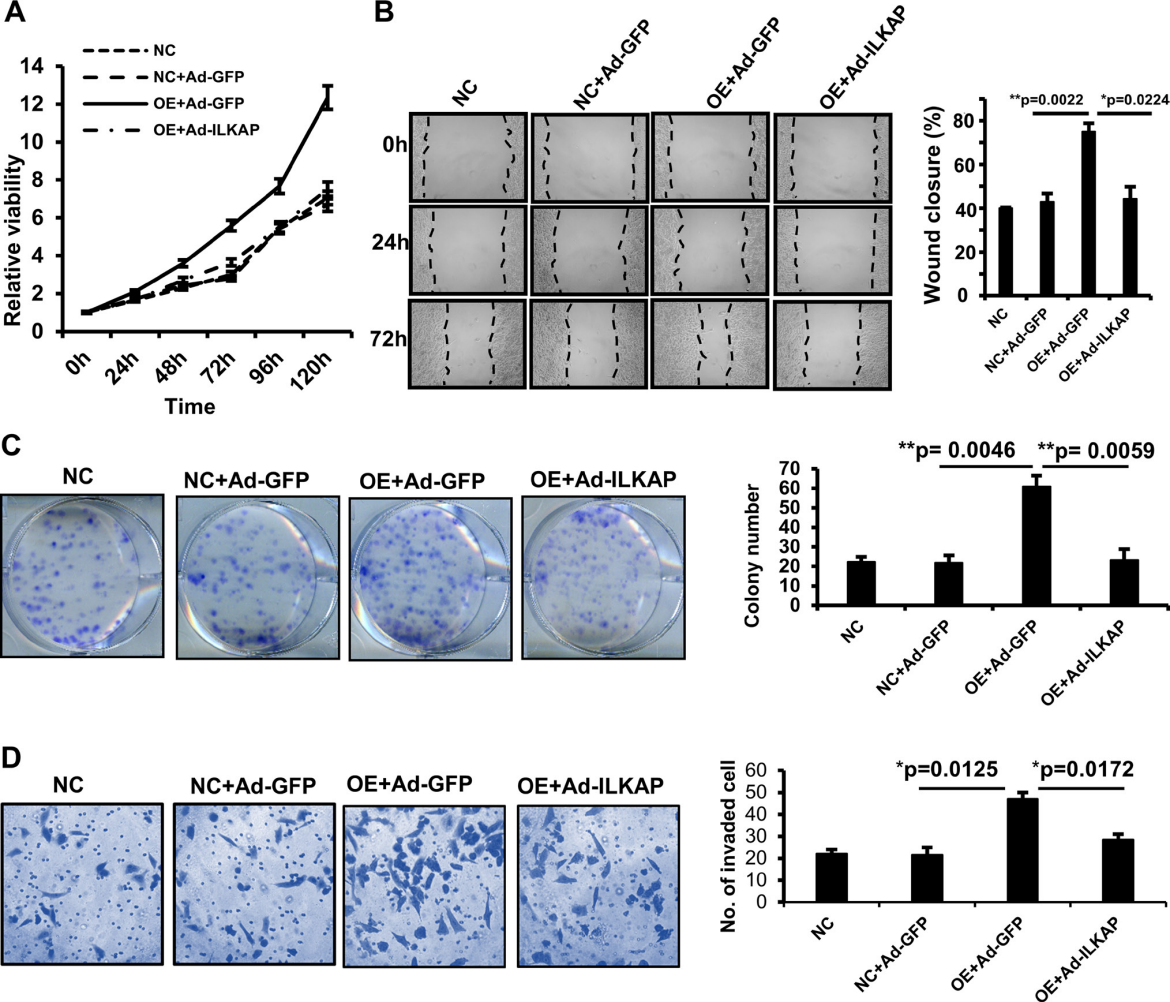
Adenovirus-mediated *ILKAP* overexpression suppresses the oncogenic roles of *MAEL* The GES-1 cells stably expressing *MAEL* were infected with adenovirus expressing *GFP* or *ILKAP*. (**A**) Dynamic changes of cell viability determined by MTT assay. The cells were harvested for MTT assay at different time after adenovirus infection. (**B**) Representative images (left panel) of wound area at the indicated time and percentage of wound closure at 72 h after scratching (right panel). (**C**) Representative images of colony (left panel) and the colony numbers (right panel). (**D**) Representative images (left panel) and statistical analysis (right panel) of invaded cells in the Transwell invasion assay. Mock: GES-1 cells, NC: GES-1 cells stably expressing empty vector; OE: GES-1 cells stably expressing *MAEL* cDNA, Ad-GFP: adenovirus expressing *GFP*, Ad-ILKAP: adenovirus expressing *ILKAP*. All the values were presented as means ± S.D for at least three independent *in vitro* experiments. Differences between two groups were analyzed by student *t*-test; ^*^p-value ≤ 0.05 and ^**^p-value ≤ 0.01.

ILKAP has been found to play tumor suppressive roles by catalyzing the dephosphorylation of some oncogenic kinases, such as RSK2 [27]. To confirm whether the oncogenic role of *MAEL* is mediated by decreasing ILKAP protein levels, we investigated the influence of *ILKAP* rescue on the oncogenic roles of *MAEL*. GES-1 cells stably expressing *MAEL* were generated via a lentivirus-mediated expression system and then infected with adenoviruses expressing ILKAP or GFP. As shown in Figure 7, *MAEL* overexpression promoted cell proliferation, colony formation, cell migration and invasion, while adenovirus-mediated *ILKAP* overexpression reversed the oncogenic transformation induced by *MAEL* overexpression in GES-1 cells. *MAEL* has been found to be involved in the process of epithelial-mesenchymal transition (EMT) [11, 12]. We investigated the effects of *MAEL* on an epithelial marker E-cadherin and a mesenchymal marker N-cadherin levels, and found that *MAEL* overexpression suppressed the E-cadherin expression and induced N-cadherin expression, whereas *ILKAP* overexpression abrogated the effects of *MAEL* on expression of both E-cadherin and N-cadherin (Supplementary Figure 4C). We further confirmed influence of *ILKAP* rescue on the oncogenic roles of *MAEL* in GC cell line BGC-823 (Supplementary Figure 5). To evaluate the *in vivo* effects of *ILKAP* and *MAEL* on tumor growth, a xenograft tumor model was generated. When the tumor volumes reached 50–100 mm^3^, adenoviruses expressing *ILKAP* or *GFP* were injected into the tumors. Based on our results, injection of adenoviruses expressing *ILKAP* significantly suppressed the growth of xenograft tumors derived from HGC-27 cells (Supplementary Figure 6). Taken together, these data demonstrated that *ILKAP* suppresses the oncogenic roles of *MAEL*.

## DISCUSSION

The cancer-testis gene *MAEL* has been found to exert oncogenic effects in liver, bladder and colorectal cancers [11–13], but anti-oncogenic effects in ovarian cancer [15]. In this study, we found that *MAEL* mRNA was expressed at higher levels in gastric cancer, glioblastoma, invasive breast cancer and lung adenocarcinoma than in their corresponding normal tissues (fold change ≥2, *p*-value < 0.05) by online analysis of transcriptomic datasets from cancer patients on the Oncomine platform. Kaplan-Meier Plotter analysis revealed that high *MAEL* mRNA levels predicted poor survival in GC patients but was not associated with overall survival in breast, lung and ovarian cancers. Furthermore, silencing *MAEL* expression in GC cells suppressed cell proliferation, colony formation, migration, invasion and the growth of xenograft tumors, while its overexpression exerts the opposite effects in normal gastric epithelial cells. These findings strongly suggest that *MAEL* plays an oncogenic role in GC progression.

Most cancer-testis genes are activated by DNA hypomethylation in cancer cells [5], and *MAEL* expression has been reported to be regulated by DNA methylation in breast and colorectal cancer [10, 28]. According to our analysis of the transcriptomics and epigenomics data from TCGA database using the cBioPortal tool, *MAEL* expression is strongly negatively correlated with DNA methylation status (Pearson’s *r* = −0.675) in GCs. We verified the CpG island methylation of *MAEL* gene promoter among gastric patient specimens and cell lines using pyrosequencing, the result was consistent with that of cBioPortal analysis. Moreover, treatment with demethylating agents enhanced *MAEL* expression, suggesting that high *MAEL* mRNA level may result from DNA hypomethylation in gastric cancer.

In consistent with the studies in liver, bladder and colorectal cancers [11–13], we demonstrated that MAEL promote tumor growth and progression in GCs. However, the underlying mechanism is not exactly the same in different types of cancer. In liver cancer, MAEL play roles by activation of Akt/GSK-3b/Snail signaling [11]; in colorectal cancer, MAEL and Snail synergistically inhibits the transcription of E-cadherin [12]; MAEL enhances the binding of DNMT3B to the promoter of MTSS1, and thereby epigenetically suppressing the MTSS1 transcription in bladder cancer [13]. Here, we demonstrated that MAEL negatively regulated ILKAP protein levels by promoting its lysosome-dependent degradation in GC cells, while treatment with the proteasome inhibitor MG132 has no significant effect on the MAEL-mediated degradation of ILKAP protein (Figure 5F and Supplementary Figure 7). ILKAP is a member of the PP2C phosphatase family. It functions as a tumor suppressor [27, 29, 30], is down-regulated in malignant melanoma [31] and is deleted in oral cancer [32]. Based on our findings, *MAEL* overexpression contributes to the depression of *ILKAP* in gastric cancer. Moreover, rescuing *ILKAP* can counteract the influence of *MAEL* overexpression on cell proliferation, colony formation, migration and invasion. These data suggest that *MAEL* exerts oncogenic effects in an ILKAP-dependent way.

The first identified substrate of ILKAP was ILK1, which promotes anchorage-independence, motility, apoptosis and angiogenesis. ILK1 has been reported to mediate the phosphorylation of PKB on Ser473 and GSK3β on Ser9, while ILKAP selectively inhibits ILK-mediated GSK3β phosphorylation, but does not affect AKT phosphorylation [25, 26]. Given that MAEL enhances ILKAP degradation, it should induce GSK3β phosphorylation. However, we did not observe any effects by MAEL on GSK3β or AKT phosphorylation. This result is consistent with a study in bladder cancer [13]. In contrast, Liu *et al.* demonstrated that MAEL increased AKT and GSK3β phosphorylation in hepatocellular carcinoma [11]. It is possible that *MAEL* regulates different pathways in different cancer types. ILKAP has also been found to dephosphorylate RSK2, decrease the expression of its substrate cyclin D1, and then induce cell apoptosis [27]. Therefore, we investigated the effects of MAEL on RSK2 phosphorylation. As expected, *MAEL* knockdown in gastric cells increased ILKAP levels and decreased RSK2 phosphorylation levels, while *MAEL* overexpression decreased ILKAP protein levels and induced RSK2 phosphorylation. Moreover, adenovirus-mediated *ILKAP* overexpression significantly reversed MAEL-induced RSK2 phosphorylation. This suggests that MAEL suppresses the ILKAP-mediated dephosphorylation of RSK2. Kim *et al.* identified p38 and CHK1 as potential ILKAP substrates using an affinity pulldown assay [24]. However, it is unknown whether ILKAP dephosphorylates p38 and CHK1/2 *in vivo*. Here, we showed that MAEL increased p38 and CHK1 phosphorylation and that the MAEL-induced phosphorylation of p38 and CHK1 can be attenuated by ILKAP. RSK2, CHK1 and p38 have been reported to be associated with tumor growth, survival and chemotherapy resistance. Constitutive activation of p38 has been reported as a major causative factor of carcinogenesis and chemotherapy resistance in GC [33, 34]. CHK1 ablation by small interfering RNAs significantly inhibits cell proliferation and sensitizes the effects of ionizing radiation (IR) treatment for GC [35]. Based on the above findings, *MAEL* exerts oncogenic effects by promoting ILKAP degradation and activating RSK2, CHK1 and p38.

In summary, we have demonstrated that *MAEL* expression may serve as an unfavorable prognostic marker for GC patients and defined a novel oncogenic pathway for *MAEL*. Ectopic expression of *MAEL* stimulates the phosphorylation and activation of p38, CHK1 and RSK2 by inducing the degradation of the phosphatase ILKAP, which facilitates tumor growth and metastasis. Moreover, in a xenograft tumor mouse model, we showed that silencing *MAEL* expression or intratumorally injecting adenoviruses expressing ILKAP suppresses tumor growth. Therefore, upregulating *ILKAP* expression and interfering with *MAEL* expression may be useful gene therapy strategies for GC.

## MATERIALS AND METHODS

### Online analysis

The differential expression of *MAEL* mRNA between cancerous and noncancerous tissues was analyzed using the microarray datasets in Oncomine (http://www.oncomine.org), a cancer microarray database and integrated data-mining platform [36]. In this study, we used the NCBI GEO dataset GSE27342 for analysis of *MAEL* mRNA expression, which includes transcriptomic data of paired tumor and adjacent normal tissues from 80 gastric cancer patients [17]. Kaplan-Meier survival curves were calculated and plotted using the Kaplan-Meier Plotter online tool (http://kmplot.com) [21], which includes gene expression data and survival information of a total of 1,051 GC patients derived from NCBI GEO datasets GSE14210 (*n* = 146), GSE15459 (*n* = 200), GSE22377 (*n* = 43), GSE29272 (*n* = 268), GSE51105 (*n* = 94), and GSE62254 (*n* = 300). All the above six datasets were used for exploring prognostic role of MAEL mRNA expression in GC. The cBioPortal [37] tool (http://www.cbioportal.org) was used to analyze the correlation between gene expression and copy number/methylation based on the cancer genomics data (Stomach Adenocarcinoma, TCGA, Provisional), which includes mRNA expression, DNA methylation, copy number variation, somatic mutation data of 478 gastric adenocarcinoma tissue cases.

### Cell lines, plasmids, antibodies and chemicals

The human embryonic kidney cell line HEK293, gastric epithelial cell lines (GES-1 and RGM1) and GC cell lines (AGS, HGC-27, KATO-III, SUN-1, N87, and BGC-823) were purchased from ATCC (Manassas, VA, USA). The Myc-tagged *MAEL* expression construct Myc-MAEL was previously described [22]. The Myc-MAEL plasmid was used a template to construct the deletion mutants of *MAEL* by overlapping PCR. The *ILKAP* coding sequence was cloned into the pCMV-HA vector at the SalI and KpnI sites, generating the HA-tagged *ILKAP* expression construct HA-ILKAP. The antibodies and chemicals used in this study are shown in Supplementary Tables 1 and 2.

### Tissue specimens

Gastric cancer and adjacent noncancerous tissues were collected from The First Affiliated Hospital, Hunan Normal University. The samples were histologically confirmed by staining with hematoxylin and eosin. The clinicopathological parameters of the gastric cancer patients were listed in Supplementary Table 3. This study was approved by the Ethics Committee of Hunan Normal University.

### Generation of stable cell lines

The lentiviral particles expressing the *MAEL* coding sequence or empty vector (GeneChem, Shanghai, China) were transduced into the gastric epithelial cell line GES-1, and puromycin was used for the selection and maintenance of the stable cells. The lentiviral particles expressing *MAEL* shRNA or scrambled shRNA (GeneCopoeia, Guangzhou, China) were used to infect the AGS and HGC-27 GC cell lines. The target sequence (GGAACTGGCCACCTATCTACT) for *MAEL* was described previously by Li *et al.* [13]. After infection, the cells were selected with hygromycin B.

### Methylation analysis by pyrosequencing

The DNA methylation status of MAEL gene promoter was analyzed using pyrosequencing assay as described by Kim *et al.* [28]. Genomic DNA was processed with EpiTect Bisulfite conversion kit (Qiagen, Valencia, CA, USA) according to the manufacturer’s instruction. The bisulfite-modified DNA was amplified using a forward primer and a biotinylated reverse primer. Amplification was performed in the Veriti 96-well thermal cycler (Qiagen) as follows: a pre-incubation at 95°C for 3 min, followed by 50 cycles of 95°C for 15 sec, 52°C for 20 sec and 72°C for 30 sec, and a final extension at 72°C for 5 min. Pyrosequencing reactions were carried out with a sequencing primer on the PyroMark Q96 system (Qiagen) according to the manufacturer’s specifications. The primers for amplification and pyrosequencing were described previously by Kim *et al.* [28]. The sequence to be detected by pyrosequencing is CGCGGAAACACCCGC, which includes 3 CpG dinucleotides (Supplementary Figure 2). An overall *MAEL* promoter methylation level was calculated as the average of the proportions of C (%) at the 3 CpG sites. SigmaPlot (Systat Software, San Jose, CA) was used for graphing and statistical analysis of correlation between mRNA level and methylation.

### RNA preparation, cDNA synthesis and real-time PCR

Total RNA was extracted from cell lines or tissues using TRIzol reagent (Invitrogen, Carlsbad, CA, USA), and then reverse transcribed into cDNA using M-MLV RTase and a random primer (GeneCopoeia, Guangzhou, China). SYBR green-based realtime PCR was performed ABI 7900 thermocycler (Thermo Fisher Scientific, MA, USA). The primers were described previously [10].

### Adenovirus transduction

The adenoviruses expressing ILKAP or GFP (Obio Technology Corp, Shanghai, China) were infected into cells at 50 multiplicity of infection (MOI).

### Xenograft tumor model

BALB/c nude mice (7–8 weeks old) were purchased from SLACCAS Jingda (Changsha, China). A total of 200 μL of serum-free DMEM containing 5 × 10^6^ cells (HGC-27 cells stably expressing the scrambled or *MAEL* shRNAs) or 1 × 10^7^ cells (GES-1 cells stably expressing the empty vector or *MAEL*) was injected into the back of nude mice. The length and width of the tumors were measured by a vernier caliper every 3 days, and the tumor volume was calculated by the following formula: volume = width^2^ × length/2. The experiments were approved by the Animal Care and Use Committee of Hunan Normal University, and all animals were handled in accordance with the guidelines of the Hunan Provincial Council on Animal Care.

### Co- IP and western blotting

Co-IP and Western blotting were performed as described previously [38]. Briefly, HEK293 cells were co-transfected with HA-ILKAP plasmid and Myc-tagged wildtype or deletion mutants of *MAEL* and harvested at 24 h post-transfection. Cell lysates were prepared and immunoprecipitated with rabbit anti-HA/Myc antibody or pre-immune rabbit IgG, and the precipitated proteins were detected by Western blot analysis using murine anti-Myc and anti-HA antibodies. For co-IP of endogenous ILKAP and MAEL proteins, lysates of AGS cells were immunoprecipitated with mouse anti-ILKAP antibody or pre-immune rabbit IgG, and the precipitated proteins were detected by Western blot analysis using rabbit anti-MAEL and mouse anti-ILKAP antibodies.

### MTT cell proliferation

The cells were cultured in serum-free medium for 24 h and then seeded in 96-well plates with normal medium for the indicated times. Cell viability was determined by the MTT assay as described previously [39].

### Wound-healing and invasion assays

For wound-healing assays, cells were cultured in a 24-well plate until they reached 90% confluency and were subsequently serum-starved overnight. After scratching, the cells were cultured in medium with a low concentration of serum (1%) until complete closure of the scratch wound was observed. Photos were obtained at different time points using an inverted microscope. Invasion assays were performed using a Corning Matrigel invasion chamber (Tewksbury, MA, USA), which consists of a cell culture insert with an 8-µm pore size PET membrane uniformly coated with Matrigel matrix.

### Colony formation assay

Cells were cultured in 6-well plates until colonies became visible to the naked eye. The colonies were subsequently rinsed with PBS, fixed with methanol and stained with Giemsa.

### Statistical analysis

All the statistical analyses were performed with Excel 2010 (Microsoft, Seattle, WA, USA). The values are presented as the mean ± S.D. Differences between two groups were analyzed with Student’s *t*-test. The level of statistical significance is expressed as the *p*-value; ^*^p-value ≤ 0.05 and ^**^p-value ≤ 0.01.

## SUPPLEMENTARY MATERIALS FIGURES AND TABLES


