# Supplementary material for: MAEL contributes to gastric cancer progression by promoting ILKAP degradation
Source: Oncotarget. 2017 Dec 6;8(69):113331–44. doi: 10.18632/oncotarget.22970 (PMC5768331; doi:10.18632/oncotarget.22970)
Supplement: Supplementary file 1 [file oncotarget-08-113331-s001.pdf]

# MAEL contributes to gastric cancer progression by promoting ILKAP degradation

## SUPPLEMENTARY MATERIALS

**Supplementary Table 1: Antibodies used in the study**

| Antibodies against | Catalog No | Source |
|---|---|---|
| MAEL | PA5-20923 | ThermoFisher, Rockford, IL, USA |
| ILKAP | HPA004752 | Sigma-Aldrich, St.Louis, MO, USA |
| ILKAP | H00080895-B01P | Novus Biologicals, Littleton, CO, USA |
| p38 MAPK | 8690 | CST, Danvers, MA, USA |
| phospho-p38 (T180/T182) | 4511 | CST, Danvers, MA, USA |
| phospho-RSK2 (S227) | 3556 | CST, Danvers, MA, USA |
| phospho-Chk1(S345) | 2348 | CST, Danvers, MA, USA |
| Phospho-GSK-3β (S9) | 5558 | CST, Danvers, MA, USA |
| Phospho-Akt (S473) | 4060 | CST, Danvers, MA, USA |
| E-cadherin | 3195 | CST, Danvers, MA, USA |
| N-cadherin | 13116 | CST, Danvers, MA, USA |
| β-Actin | AT0001 | CMCTAG, Milwaukee, WI, USA |
| GAPDH | AT0002 | CMCTAG, Milwaukee, WI, USA |
| Myc-tag | C3956 | Sigma-Aldrich, St.Louis, MO, USA |
| HA-tag | SAB1305536 | Sigma-Aldrich, St.Louis, MO, USA |
| HRP-labeled goat α-rabbit IgG | 074-1506 | KPL, Gaithersburg, MD, USA |
| HRP-labeled goat α-mouse IgG | 5220-0287 | KPL, Gaithersburg, MD, USA |

**Supplementary Table 2: Chemicals used in the study**

| Chemicals | Source |
|---|---|
| hygromycin B | BBI, Shanghai, China |
| puromycin | BBI, Shanghai, China |
| Leupetin | BBI, Shanghai, China |
| Chloroquine | BBI, Shanghai, China |
| MG132 | BBI, Shanghai, China |
| 3-MA | BBI, Shanghai, China |
| 5-aza-2-deoxycytidine | Sigma-Aldrich, St.Louis, MO, USA |
| MTT | BBI, Shanghai, China |

**Supplementary Table 3: The clinicopathological parameters of the gastric cancer patients used in the study**

| Gender | Age | Pathology | Differentiation | Diameter | TNM | Stage |
|---|---|---|---|---|---|---|
| Male | 86 | adenocarcinoma | poor | 4 cm | T3N1M1 | III |
| Male | 55 | adenocarcinoma | high | 1 cm | T1N0M0 | I |
| Female | 48 | adenocarcinoma | poor | 2.5 cm | T3N0M0 | II |
| Male | 68 | adenocarcinoma | poor | 4 cm | T3N0M1 | II |

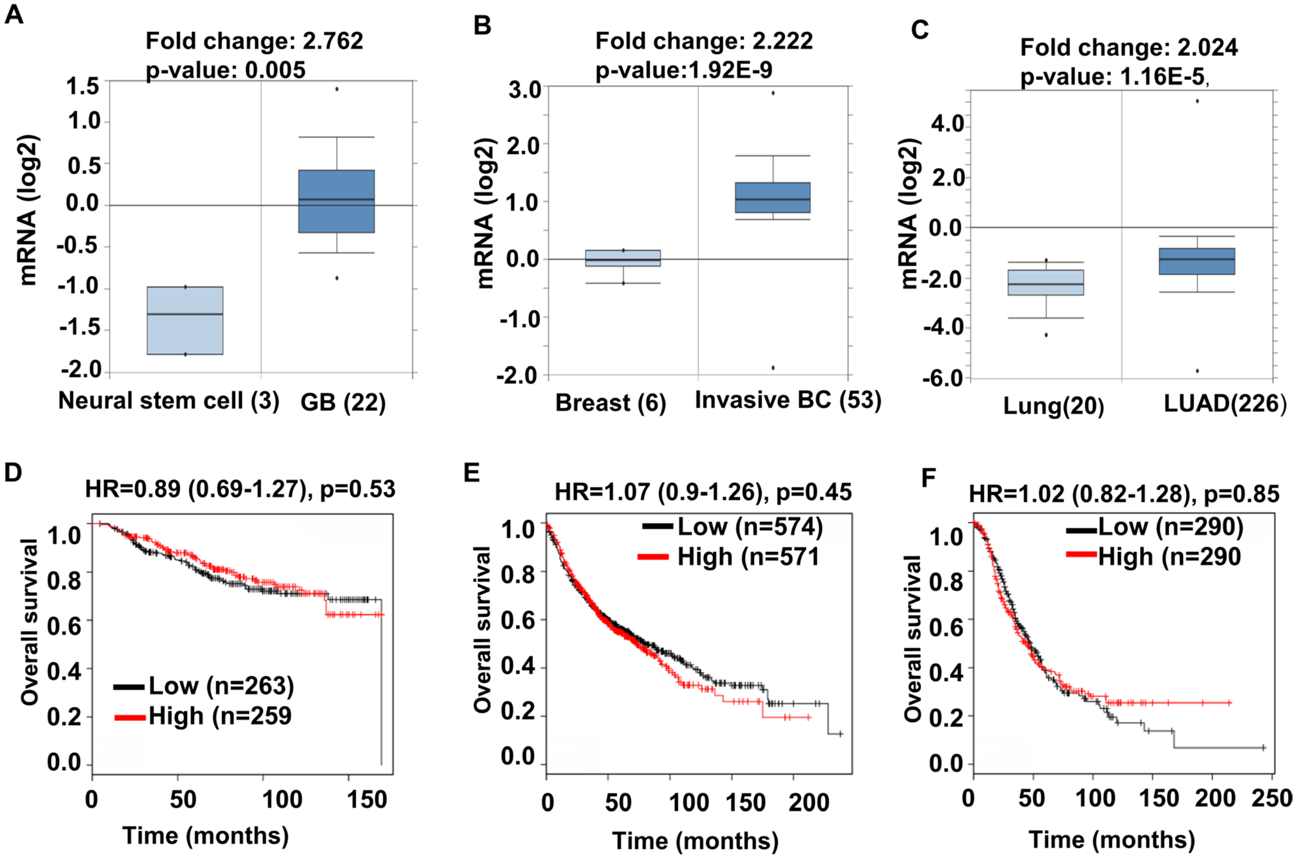


**Supplementary Figure 1: Expression and prognostic significance of *MAEL* gene.** (**A–C**) Differential expression of *MAEL* mRNA in normal and cancer tissues were analyzed using Onocomine based on the NCBI GEO datasets (glioblastoma: GSE4536, invasive breast cancer: GSE9014 and lung adenocarcinoma: GSE31210). GB: glioblastoma, BS: breast cancer, OS: overall survival, LUAD: lung adenocarcinoma. (**D–F**) The survival curves of patients with breast (**D**), lung (**E**) and ovarian (**F**) cancers were plotted using Kaplan Meier plotter, which includes 5,143 breast, 1,816 ovarian, 2,437 lung and 1,065 gastric cancer patients with a mean follow-up of 69/40/49/33 months.

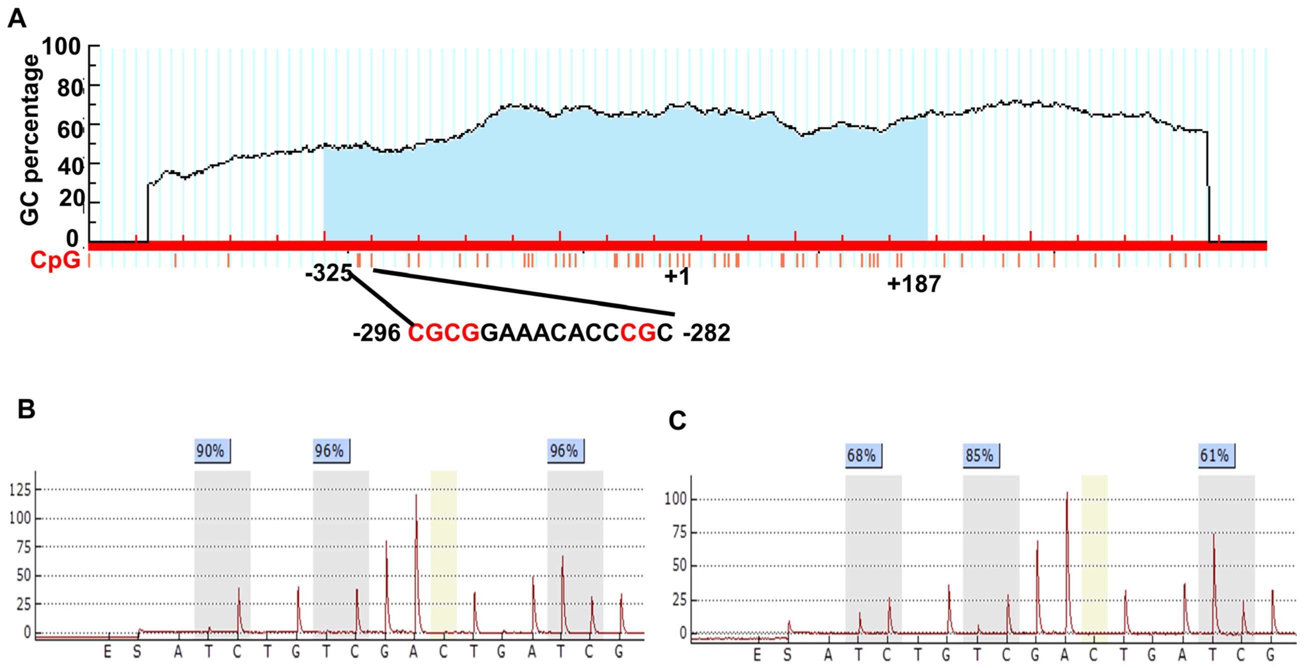


**Supplementary Figure 2: Methylation level of *MAEL* gene promoter was quantified by pyrosequencing.** (**A**) Schematic diagram of the *MAEL* gene promoter. The CpG island (blue) was predicted by MethPrimer software, which locates at nucleotides −293 to +178, relative to transcription start site (+1). The sequence to be detected by pyrosequencing is CGCGGAAACACCCGC, which includes 3 CpG dinucleotides. (**B**, **C**) The pyrograms of an adjacent normal gastric tissue (**B**) and a gastric tumor tissue (**C**). Grey areas indicate the CpG sites that were analyzed. The percentages (blue) are the proportion of C at each CpG site after bisulfite conversion. The yellow regions indicate controls regions for automatic assessment of bisulfite conversion (unmethylated C should be fully converted to T). An overall *MAEL* promoter methylation level is calculated as the average of the proportions of C (%) at the 3 CpG sites. The y-axis represents the signal intensity, the x-axis shows the dispensation sequence.

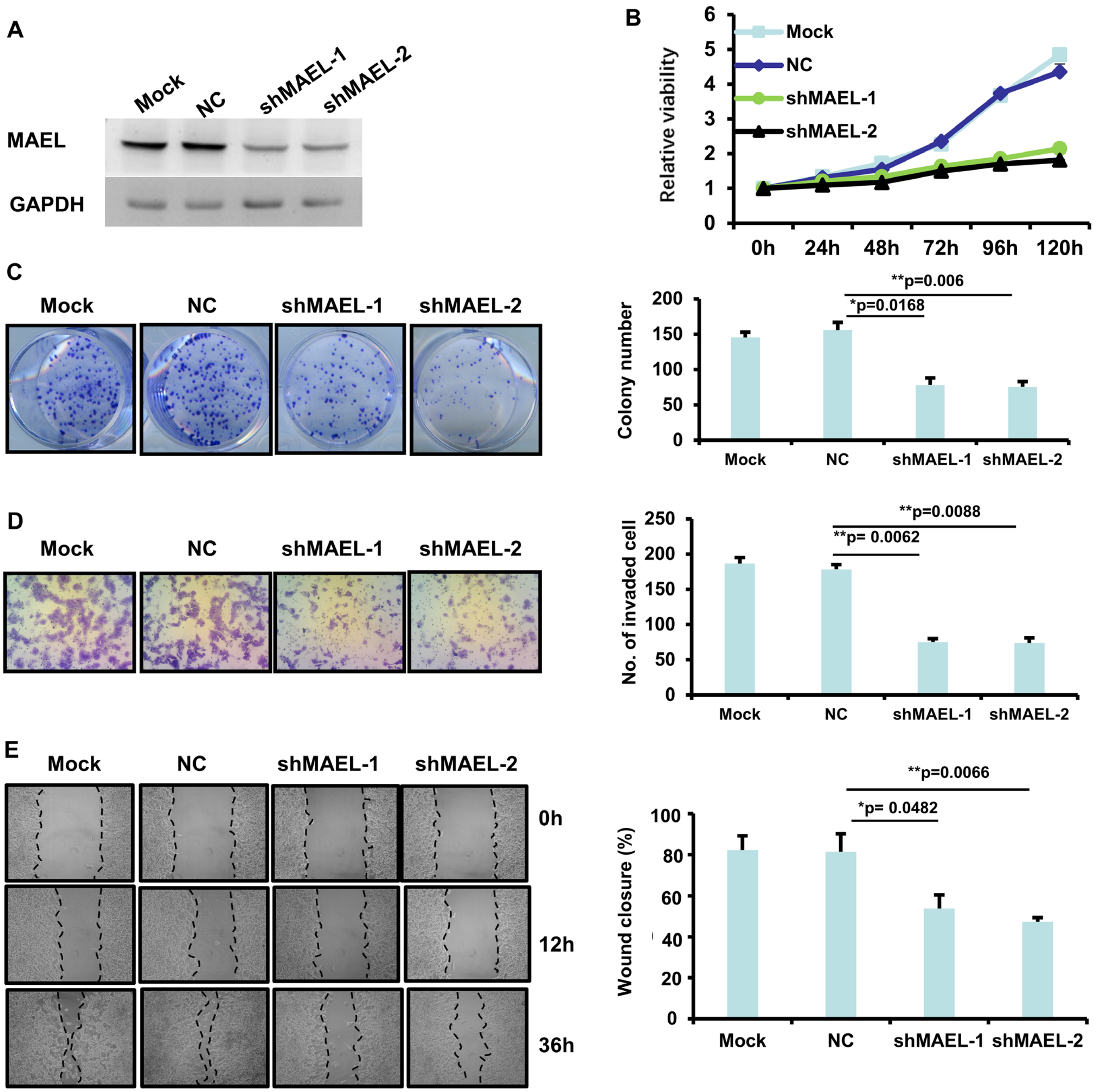


**Supplementary Figure 3: *MAEL* knockdown inhibits proliferation, migration and invasion in SUN-1 cells.** (**A**) MAEL protein levels in the untreated SUN-1 cells (mock) and the SUN-1 cells stably expressing *MAEL* shRNA (shRNA-1, shRNA-2) or scrambled shRNA (NC). (**B**) The cell growth was determined by MTT assay. (**C**) Representative images of colony (left panel) and the colony numbers (right panel). (**D**) Representative images (left panel) and statistical analysis (right panel) of invaded cells in the Transwell invasion assay. (**E**) Representative images of wound area (left panel) at the indicated time and percentage of wound closure (right panel) at 36 h after scratching. The target sequences of MAEL for constructing lentiviral shRNA were as follows: shRNA1: 5′-GGAACTGGCCACCTATCTACT-3′; shRNA2: 5′-GAGTCAACTGGTGTTTGAAGC-3′. All the values were presented as means ± S.D for at least three independent *in vitro* experiments. Differences between two groups were analyzed by student *t*-test; $^{*}p$-value ≤ 0.05 and $^{**}p$-value ≤ 0.01.

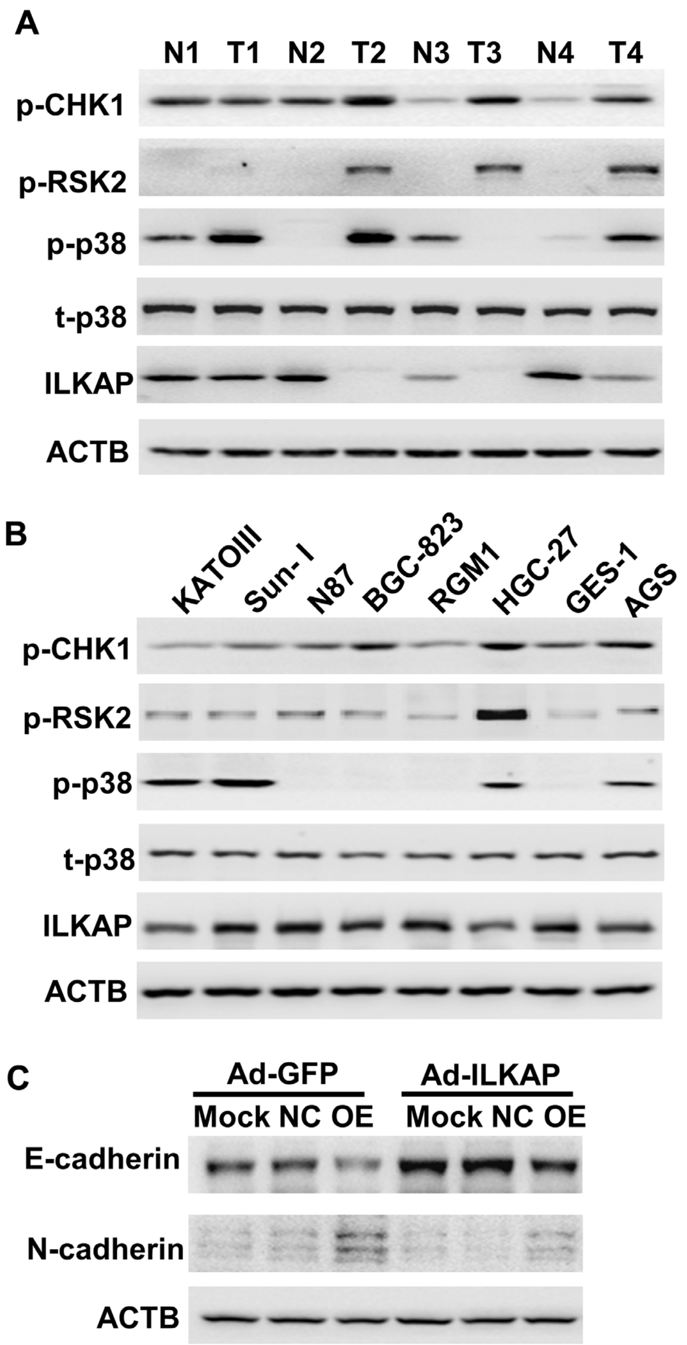


**Supplementary Figure 4: Western blot analysis.** (**A**) The expression of ILKAP and phosphorylated CHK1, RSK2, p38 in the 4 pairs of gastric tumor and adjacent normal tissues. (**B**) The expression of ILKAP protein and phosphorylated CHK1, RSK2, p38 in the gastric cell lines. (**C**) Effect of adenovirus-mediated ILKAP expression on the levels of E-cadherin and N-cadherin. The GES-1 cells (mock) and the GES-1 cells stably expressing empty vector (NC) or MAEL cDNA (OE) were infected with adenovirus expressing *GFP* or *ILKAP*. At 48 h after infection, the cells were harvested and subjected to Western blotting with indicated antibodies.

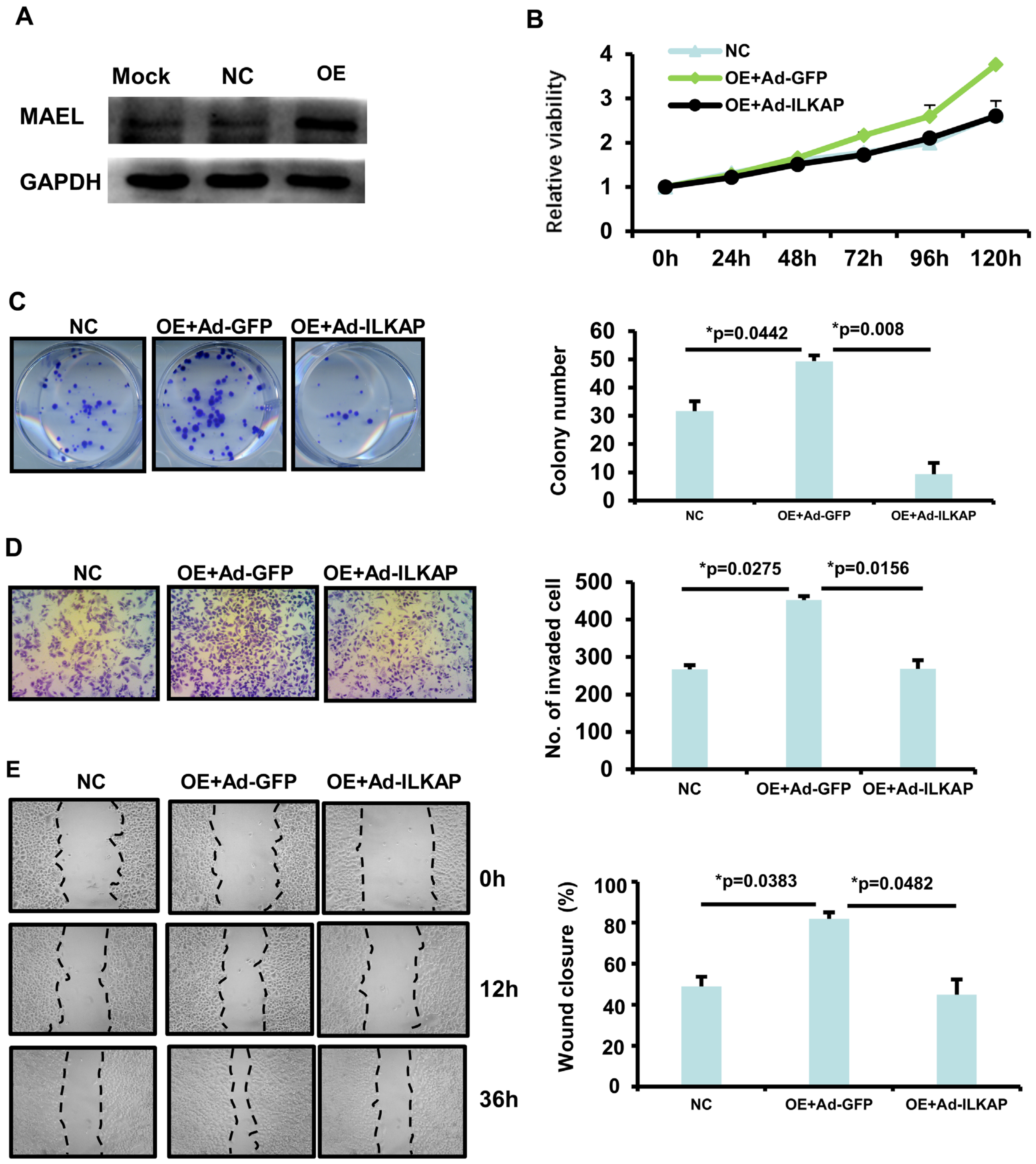


**Supplementary Figure 5: Adenovirus-mediated *ILKAP* overexpression suppresses the oncogenic roles of *MAEL*.** The BGC-823 cells stably expressing *MAEL* cDNA or empty vector were infected with adenovirus expressing GFP (Ad-GFP) or ILKAP (Ad-ILKAP). (**A**) The *MAEL* expression levels in BGC-823 cells (Mock) and the BGC-823 cells with stable expression empty vector (NC) or *MAEL* cDNA (OE) were detected by Western blotting. (**B**) Dynamic changes of cell viability determined by MTT assay. The cells were harvested for MTT assay at different time after adenovirus infection. (**C**) Representative images of colony (left panel) and the colony numbers (right panel). (**D**) Representative images (left panel) and statistical analysis (right panel) of invaded cells in the Transwell invasion assay. (**E**) Representative images (left panel) of wound area at the indicated time and percentage of wound closure at 36 h after scratching (right panel). All the values were presented as means ± S.D for at least three independent *in vitro* experiments. Differences between two groups were analyzed by student *t*-test; $^{*}p$-value ≤ 0.05 and $^{**}p$-value ≤ 0.01.

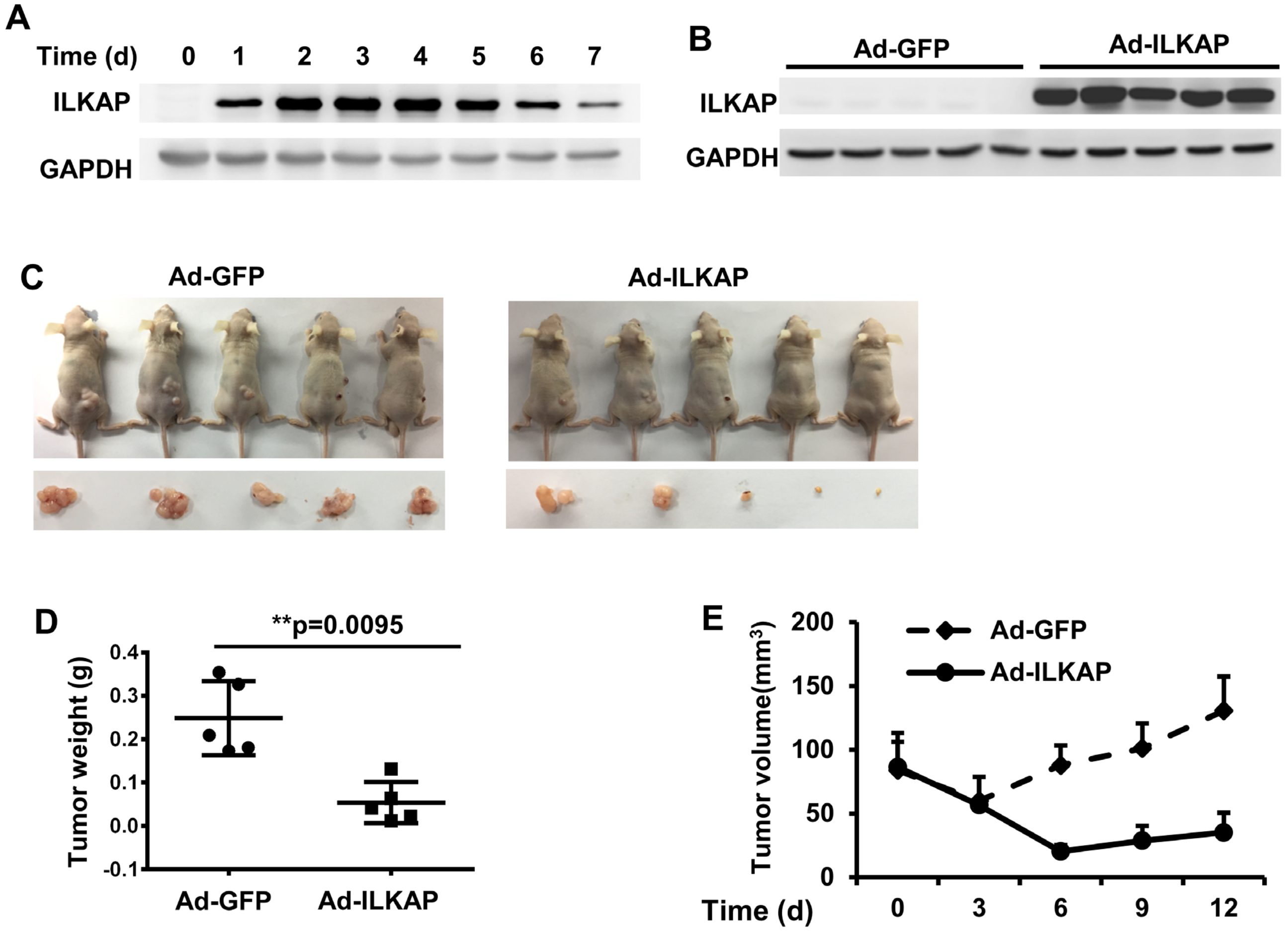


**Supplementary Figure 6: Therapeutic effects of adenoviruses expressing ILKAP on xenograft tumors.** (**A**) Dynamic changes of ILKAP protein levels in the HGC-27 cells after infection with adenoviruses expressing *ILKAP*. (**B–D**) The therapeutic effects of adenovirus-mediated ILKAP expression. BALB/c nude mice (7–8 weeks old) were injected with $5 \times 10^6$ HGC-27 cells at the back of nude mice. When the tumor volume reached 50–100 $mm^3$, the adenovirus expressing *ILKAP* or *GFP* were injected intratumorally at a dose of $1 \times 10^9$ PFU (pore-forming unit) in 100 μL of phosphate-buffered saline (PBS). Mice received a second injection at 2 days after the first injection. (**B**) Expression of ILKAP protein in the xenograft tumors injected with Ad-GFP or Ad-ILKAP at 48 h after injection. (**C**) Representative images of the xenograft tumors derived from HGC-27 cells at 15 days after adenovirus injection. (**D**) Weights of xenograft tumors at 15 days after adenovirus injection. (**E**) Dynamic volume of xenograft tumors at different time after adenovirus injection.

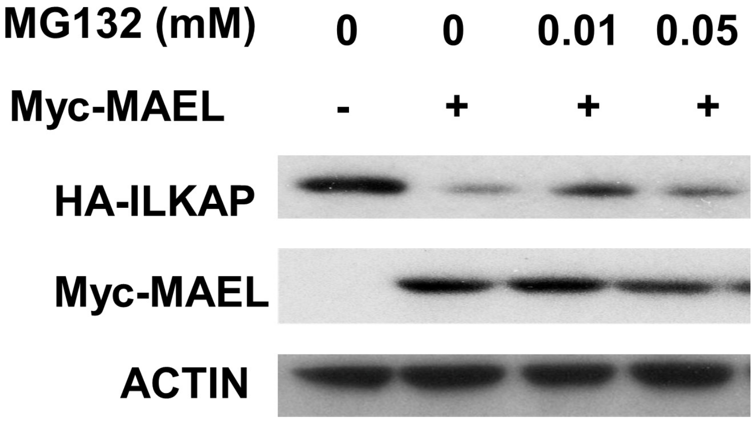


**Supplementary Figure 7: Treatment with MG132 has no significant effect on ILKAP levels.** The HEK293 cells were transfected with HA-ILKAP and Myc-MAEL plasmids and treated with CHX plus MG132 for 4 h, HA-ILKAP protein levels were detected by Western blotting.
